# The diversity and abundance of bacterial and fungal communities in the rhizosphere of *Cathaya argyrophylla* are affected by soil physicochemical properties

**DOI:** 10.3389/fmicb.2023.1111087

**Published:** 2023-06-12

**Authors:** Peng Xie, Kerui Huang, Aihua Deng, Ping Mo, Fen Xiao, Fei Wu, Dewei Xiao, Yun Wang

**Affiliations:** ^1^College of Life and Environmental Sciences, Hunan University of Arts and Science, Changde, Hunan, China; ^2^College of Agriculture, Forestry and Technology, Hunan Applied Technology University, Changde, Hunan, China; ^3^Central South University of Forestry and Technology Changsha, Hunan, China; ^4^Qingjie Mountain State Forest Farm, Chengbu, Hunan, China; ^5^Chukou State-Owned Forest Farm, Zixing, Hunan, China

**Keywords:** endangered species, soil bacterial community, fungal diversity, natural forests, rhizosphere, soil physicochemical properties, tree growth, *Cathaya argyrophylla*

## Abstract

*Cathaya argyrophylla* is an ancient Pinaceae species endemic to China that is listed on the IUCN Red List. Although *C. argyrophylla* is an ectomycorrhizal plant, the relationship between its rhizospheric soil microbial community and soil properties related to the natural habitat remains unknown. High-throughput sequencing of bacterial 16S rRNA genes and fungal ITS region sequences was used to survey the *C. argyrophylla* soil community at four natural spatially distributed points in Hunan Province, China, and functional profiles were predicted using PICRUSt2 and FUNGuild. The dominant bacterial phyla included *Proteobacteria*, *Acidobacteria*, *Actinobacteria*, and *Chloroflexi*, and the dominant genus was *Acidothermus*. The dominant fungal phyla were *Basidiomycota* and *Ascomycota*, while *Russula* was the dominant genus. Soil properties were the main factors leading to changes in rhizosphere soil bacterial and fungal communities, with nitrogen being the main driver of changes in soil microbial communities. The metabolic capacities of the microbial communities were predicted to identify differences in their functional profiles, including amino acid transport and metabolism, energy production and conversion, and the presence of fungi, including saprotrophs and symbiotrophs. These findings illuminate the soil microbial ecology of *C. argyrophylla*, and provide a scientific basis for screening rhizosphere microorganisms that are suitable for vegetation restoration and reconstruction for this important threatened species.

## 1. Introduction

*Cathaya argyrophylla* Chunet Kuang is a Class I key protected wild plant in China due to its low population size of less than 1,000 mature individuals in the wild, resulting from severe climatic changes during the Quaternary glacial period (Qian et al., 2016; Forest et al., 2018). *C. argyrophylla* is discontinuously distributed in the subtropical mountains of Guangxi, Hunan, Sichuan, and Guizhou provinces (Fan et al., 2020). The endangered status of *C. argyrophylla* can be attributed to poor germinability, poor interspecific competition, and low adaptive ability to new environments (Xiao et al., 2022), as well as disturbances caused by broadleaf species during succession, which have led to drastic changes in genetic diversity and population structure. As the only extant species in the *Cathaya* genus, the extinction of *C. argyrophylla* would cause ecological destabilization, and could have profound effects on 10–30 species in its closely related ecosystem (Forest et al., 2018). Many scholars have conducted studies on the features of *C. argyrophylla* populations that have contributed to its endangered status, including community structure types, survival environment, reproductive patterns, and biological traits (Vaario et al., 2006; Wang and Ge, 2006; Wang et al., 2010; Xiao et al., 2022). Artificial propagation has been attempted via a seedling raising and regeneration system (Erfmeier et al., 2019; Zheng et al., 2021). Based on *in situ* and *in vitro* evidence, it has been established that *C. argyrophylla* is a typical ectomycorrhizal plant, and the microbial community within its rhizosphere is a crucial component of its ecosystem (Sun et al., 2006). The survival of *C. argyrophylla* is inextricably linked to the rhizosphere microbial communities that occur around the plant. Mycorrhizal fungi play a critical role in the survival and growth of many plants, including pine trees, by forming symbiotic relationships with their host organisms (Genre et al., 2020). Ectomycorrhizal fungi are essential in all stages of *Pinus sylvestris* var. *mongolica*, and it cannot survive independently of ectomycorrhizal fungi (Guo et al., 2020).

Since the initial colonization of land by plants approximately 400 million years ago, *Phytomycota* have evolved inextricable associations and co-evolved with their plant hosts (Brundrett, 2002). Plant-associated rhizosphere microorganisms play an indispensable role in the survival, growth, and adaptation of plants to their environment. Microorganisms fulfill integral functions in nutrient acquisition, nitrogen cycling, carbon cycling, soil morphogenesis (Wang J. et al., 2020), mineral uptake, and augmenting plant tolerance to diverse environmental stresses (Harman et al., 2020; Ge et al., 2023) including drought, salt and pathogens. Furthermore, these rhizosphere microbial communities are vital to maintain plant health by enabling nutrient sequestration, promoting drought and salt tolerance, and supporting plant flowering (Lu et al., 2018; Jiang et al., 2023; Xu et al., 2023). Different microbial communities respond differently to the host plant genotype, age, and external environmental conditions (Bahram et al., 2018; Cui et al., 2019). For example, *Variovorax* can produce growth hormones that can regulate the concentration of plant growth hormones and affect root growth (Rousk et al., 2010). Some plants have adaptations that allow them to acquire nutrients through alternative pathways under nutrient shortage circumstances; for example, inorganic and organic nutrients become available through microbial turnover of soil organic matter (Canarini et al., 2021).

The plant-associated microbial community is shaped by the host, but is also influenced by the environment (Coleman-Derr et al., 2016; Razanamalala et al., 2017). Shaping the microbial community of the root microbiome is a mechanism that many plants use to adapt to different phytogeographical regions. Plant genomes can partially determine the composition and function of a microbial community, and the plant species is therefore one of the most important factors affecting the soil microbial community (Zhang et al., 2019; Zheng and Gong, 2019; Dai et al., 2021). The characteristics of soil microbial niches can be modified by changing the physical conditions of the soil, modifying the distribution of organic matter and nutrient content, or soil temperature. Soil physicochemical properties are primary determinants of microbial diversity and community dynamics (Xie et al., 2023). Soil pH has a significant impact on microbial communities. It indirectly affects microbial survival and biodiversity by regulating factors such as organic matter solubility and redox conditions that determine microbial habitability. As a central dimension of the microbial niche, soil pH exerts system-level impacts on population dynamics and ecosystem functioning (Malik et al., 2018; Qi et al., 2018). The supply and accrual of organic carbon are key environmental determinants governing the dynamics of soil microbial communities. By providing microbes with energetic substrates, organic carbon inputs substantially reshape microbial community structure and stimulate microbial activity (Bastida et al., 2021). The plant’s short-term ability to adapt to environmental changes is driven by its closely-related and responsive microbial communities, while in the long term (over a century), plant-microbe interactions are the key driver of plant adaptation (Trivedi et al., 2022; Zhu et al., 2022). Anthropogenic climate change has had a significant impact on the habitat of *C. argyrophylla* (Ran et al., 2019), both in terms of habitat availability and fragmentation. Despite the crucial role that microorganisms play in the habitat of *C. argyrophylla*, the distribution of microorganisms among current *C. argyrophylla* habitats has not been adequately surveyed. Furthermore, the interactions between the plant and its microbiome, and their impact on plant adaptation and evolution, remain poorly understood. Investigating the microbial properties of the root zone of *C. argyrophylla*, and the factors influencing them is essential to understand how the species adapts to environmental changes. This information is also necessary to develop strategies for the conservation of *C. argyrophylla* and its ecosystem, including the mycorrhizal fungi and microbial community.

To address this issue, in the present study we collected rhizosphere soil from four distribution sites of *C. argyrophylla* in Hunan Province, China, and used high-throughput sequencing to analyze the soil physicochemical properties and microbial community structure in the rhizosphere of *C. argyrophylla*. We hypothesized that soil properties will influence the diversity and composition of bacterial and fungal communities in the *C. argyrophylla* rhizosphere. The present study aimed to (1) establish the microbial diversity and community structure of the *C. argyrophylla* rhizosphere, (2) explore the effects of physicochemical properties of the rhizosphere soil on the microbial community diversity and community structure of *C. argyrophylla*, and (3) predict the potential functions of these microbes in the ecosystem. Our findings provide new insights into the ecology of *C. argyrophylla* and its associated microorganisms, and could contribute to the development of sustainable forest management practices. These findings will also support further research toward the conservation of this critical ancient tree species and its associated microbial communities.

## 2. Materials and methods

### 2.1. Study site description

Two wild *C. argyrophylla* sites in Hunan Province were used to collect the experimental material. The first was the Shajiao Tung *C. argyrophylla* Nature Reserve, located in the Chengbu Miao Autonomous County of Hunan Province (25°58′–26°42′N, 109°58′–110°37′E). This region has a subtropical mountain climate, with a mean annual temperature of 16.1°C and a mean annual precipitation of 1,200 mm. This climate has four distinct seasons, with moderate temperatures and abundant rainfall. The wild community of *C. argyrophylla* is primarily distributed in the Luohandong forest area at the junction of Chengbu County and Xinning County in Hunan Province (26°33′N, 110°36′E). The trees are mainly concentrated in the narrow ridges of Luanyandong and Mapigu, with a higher elevation of 1,050 m and a lower elevation of 950 m. We designated the higher elevation as Shajiao Tung *C. argyrophylla* Nature Reserve No.2 (CBY) and the lower elevation as Shajiao Tung *C. argyrophylla* Nature Reserve No.1 (CBE). The soil type in this area is mountain yellow soil, and the *C. argyrophylla* community is a mixed forest type of *C. argyrophylla* and *Tsuga longibracteata*. The second site was the Bamian Mountain National Nature Reserve, which is located in the western part of Guidong County (25°54′–26°06′N, 113°37′–113°50′E). The area is characterized by a subtropical monsoon humid climate with a mean annual temperature of 15.8°C and a mean annual precipitation of 1700 mm. Rainfall has an abundant and uneven annual distribution with significant interannual variation. The wild community of *C. argyrophylla* is primarily distributed on the northwest slope of Bamian Mountain (25°57′–26°3′N, 113°40′–113°51′E), situated at the junction of Zixing and Guidong in Hunan Province. The Jiaopenliao population (JPL) of *C. argyrophylla* is located at an altitude of 1,200 m in the middle of the slope, while Simaoping (SMP) is situated at an altitude of 1,100 m in the lower part of Bamian Mountain. The soil type in this region is mountain yellow-brown soil, with a soil thickness of around 40 cm. The *C. argyrophylla* community type in this area is a mixed forest of *C. argyrophylla* and *Castanopsis eyrei*.

### 2.2. Soil sampling

Two individual sites were randomly selected from the Shajiao Tung *C. argyrophylla* Nature Reserve and the Bamian Mountain National Nature Reserve sites. Based on the topography, elevation, natural vegetation, and other characteristics of the *C. argyrophylla* distribution area, two representative sampling sites were selected in the Shajiao Tung *C. argyrophylla* Nature Reserve and two representative sampling sites were selected in the Bamian Mountain National Nature Reserve to establish standard sample plots (Table 1). Rhizosphere soil samples were obtained by gently brushing off the soil tightly adhered to the roots of *C. argyrophylla* using a sterile soft-bristled paintbrush. Soil samples were collected from the top 20 cm of the rhizosphere soil. Five independent rhizosphere soil samples were randomly collected from each plot and subsequently pooled to form a composite sample. The mixed soil samples were subdivided into two components: one part was frozen in liquid nitrogen, transported to the laboratory on dry ice, and stored at −80°C to survey the soil microbial community. The remaining sample fraction was airdried to determine the soil physicochemical properties.

**Table 1:** Geographic location of the *Cathaya argyrophylla* sampling plots.

Administrative	Sampling sites	Sampling site abbreviation	Elevation	Latitude	Longitude
Chengbu Miao Autonomous County, Shaoyang City, Hunan Province	Shajiao Tung *C. argyrophylla* Nature Reserve No.1	CBE	928 m	26°32′31.48″	110°34′48.45″
Chengbu Miao Autonomous County, Shaoyang City, Hunan Province	Shajiao Tung *C. argyrophylla* Nature Reserve No.2	CBY	1,066 m	26°32′18.37″	110°34′49.75″
Zixing County, Chenzhou City, Hunan Province	Bamian Mountain National Nature Reserve-Simaoping	SMP	1,137 m	26°4′8.88″	113°42′59.51″
Zixing County, Chenzhou City, Hunan Province	Bamian Mountain National Nature Reserve -Jiaopenliao	JPL	1,213 m	26°4′23.59″	113°42′55.92″

### 2.3. Determination of soil physicochemical properties

Soil total carbon (TC) and total nitrogen (TN) were measured using an EA 3000 elemental analyzer (Grunert et al., 2019). The pH was determined using the potentiometric method with a water-to-soil ratio of 2.5:1 (Yang et al., 2020). The available phosphorus (AP) content was determined using the hydrochloric acid ammonium chloride method. Soil organic matter (SOM) content was measured using the K_2_Cr_2_O_7_ oxidation method. Available potassium (AK) was assayed using flame photometry (Song et al., 2020). Solutions were analyzed using an FIAstar 5,000 Analyzer (Foss Tecator, Denmark) to obtain NH₄^+^-N and NO_3_^−^-N values (Huang et al., 2015). Available soil N was calculated as the sum of NH₄^+^-N and NO_3_^−^-N.

### 2.4. DNA extraction and PCR amplification

Total soil DNA was extracted from soil samples using a soil microbe DNA kit (QIAGEN, United States) according to the manufacturer’s instructions. A NanoDrop 2000 UV–vis spectrophotometer (Thermo Scientific, United States) was used to determine the concentration and purity of DNA samples, and the quality was assessed using 1% agarose gel electrophoresis. The PCR primers 338F (5′- ACTCCTACGGGAGGCAGCAG-3′) and 806R (5′- GGACTACHVGGGTWTCTAAT-3′) were used to amplify bacterial *16S* rRNA, and *ITS1F* (5′-CTTGGTCATTTAGAGGAAGTAA-3′) and *ITS2R* (5′- GCTGCGTTCTTCATCGATGC-3′) were used to amplify fungal ITS. The PCR reaction mix contained 1× FastPfu Buffer, 2.5 mM dNTPs, 5 μM each of forward and reverse primers, 1 U FastPfu polymerase (0.4 μL), and 10 ng of template DNA in a 20 μL volume. The thermocycler settings were: 2 min at 95°C, followed by 28 cycles of 45 s at 95°C, 2 min at 45°C, 3 min at 72°C, and a final elongation for 10 min at 72°C. PCR was performed in triplicate for each extracted soil sample. Amplified products were quantified using QuantiFluor™-ST (Promega, United States) and purified using the AxyPrep DNA Gel Extraction Kit according to the manufacturer’s instructions (Axygen Biosciences, USA). Sequencing was performed at Majorbio BioPharm Technology Co., Ltd. using the Illumina MiSeq platform (Illumina, San Diego, CA, United States; Shanghai, China).

### 2.5. Illumina sequencing and processing of sequencing data

To produce raw tags, 16S rRNA gene paired-end reads were assembled using Flash (v1.2.11).^1^ QIIME (v1.9.1)^2^ was used to analyze the relative abundance of taxonomic summaries, beta diversity, and rarefactions. UPARSE (v7.0.1090)^3^ was used to cluster sequences by operational taxon unit (OTU). The ribosomal database project classifier (v2.11)^4^ was used to classify taxa. USEARCH (v7.0) was used to find concatenated sequences.^5^ The classification of each 16S rRNA sequence was performed with a confidence threshold of 70%. Each 16S rRNA gene sequence was classified using the RDP classifier method,^6^ with a 70% confidence threshold, against the Silva database (Release132).^7^ Each ITS sequence was classified using the RDP classifier method, with a confidence level of 70%, against the Unite database (version 7.2).^8^ Based on this, 505,618 bacterial and 701,449 fungal sequences were obtained from 12 samples. The average length of each sequence was 410 bp, and the total number of OTUs was 2,307, which included 28 phyla, 72 classes, 167 orders, 258 families, 393 genera, and 732 species. Based The fungal ITS sequencing project enabled the clustering of reads into 590 species, 396 genera, 225 families, 110 orders, 45 classes, and 9 phyla.

### 2.6. Statistical analysis

Data means and standard deviations were computed and statistically analyzed using SPSS (SPSS, Inc., Chicago, IL, United States). Differences between groups were assessed using ANOVA and Duncan’s multiple range tests, with a *p* < 0.05 denoting statistically significant differences. The Chao1, Shannon, ACE, and Simpson indexes and rarefaction curves were computed using Mothur software [version 1.30.2, available at: https://www.mothur.org/wiki/Download_mothur (Schloss et al., 2009)], and were used to analyze the alpha diversity of the microbial community. To further explore the microbial diversity, Venn diagrams were generated at the OTU level using the Venn Diagram package (Chen and Boutros, 2011). R version 2.1.3 was used to conduct principal coordinate analysis (PCoA) to condense the original variables’ dimensions based on Bray-Curtis distances. The distinct genus distributions were determined using the linear discriminant analysis (LDA) effect size (LEfSe). An LDA score > 3.5 and *p* ≤ 0.05 were used to filter indicator genera that were considered ‘extremely enriched’. The relationship between environmental factors and microbial community structure was evaluated using the mental test method, and the significance level was set at *p* < 0.05. The functional profiles of the bacterial communities were predicted using PICRUSt2 (Langille et al., 2013) while those of the fungal communities were predicted using FUNGild (Nguyen et al., 2016). Using “pheatmap,” a Spearman correlation heatmap was generated to examine the relationship between the structures of the soil microbial community and the factors affecting the soil environment.

## 3. Results

### 3.1. Diversity of soil bacterial and fungal communities

**Figure 1:**
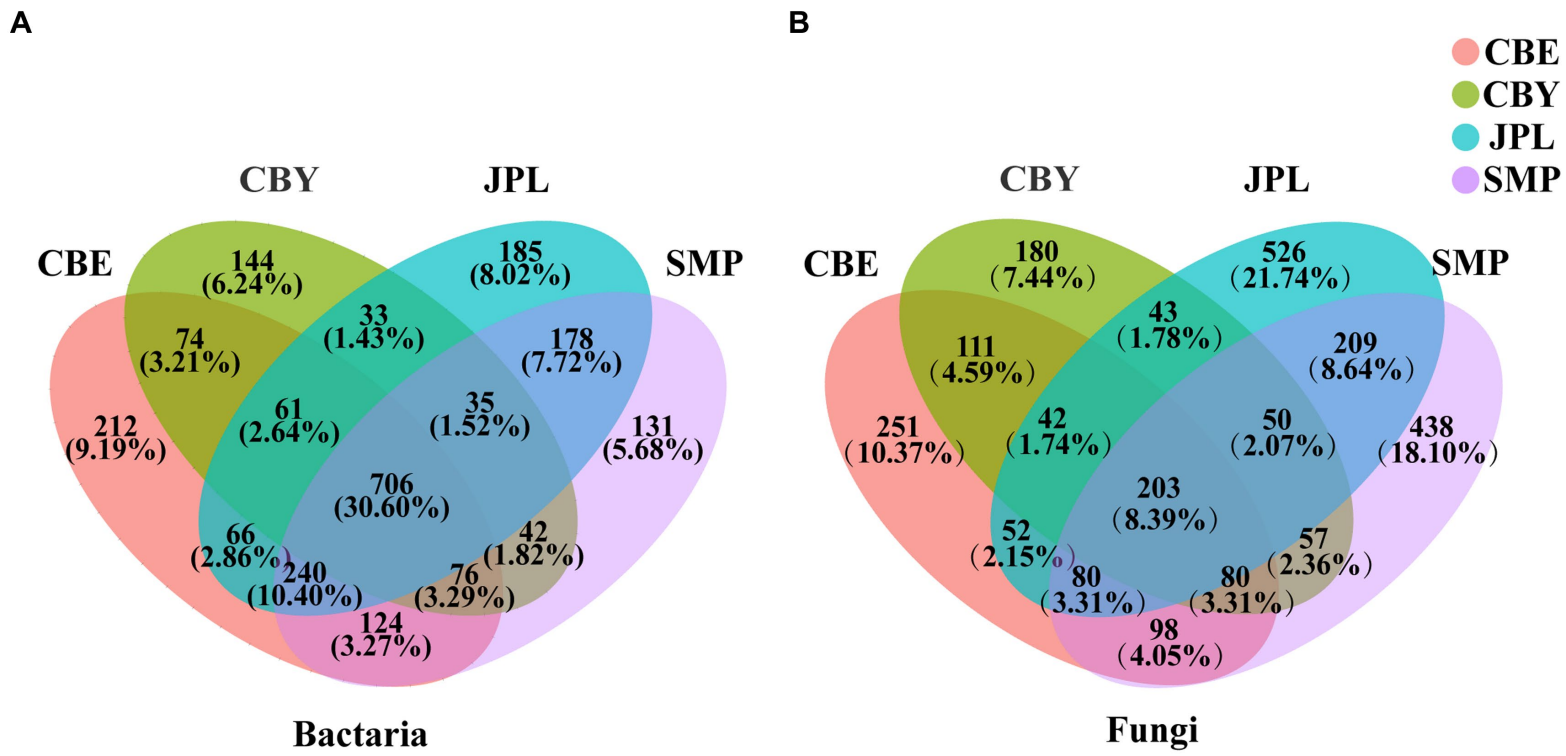
Venn diagram of microbial communities in the rhizosphere soil of *Cathaya argyrophylla* at different sites. Different colors represent distinctive groups. The numbers on each diagram refer to **(A)** the number of *C. argyrophylla* bacterial soil operational taxon units (OTUs) and **(B)** the number of *C. argyrophylla* fungal soil OTUs.

Among all samples, there was a higher proportion of OTUs shared by bacteria (30.60%) than fungi (8.39%). The bacterial OTUs were classified into the major phyla *Proteobacteria*, *Actinobacteria*, and *Acidobacteria*. CBY had the fewest OTUs for both bacteria and fungi (Figure 1). Of all OTUs, 526 (21.74%) were unique to JPL, which was significantly higher than the number of OTUs that were unique to each of the other three sampling sites.

**Figure 2:**
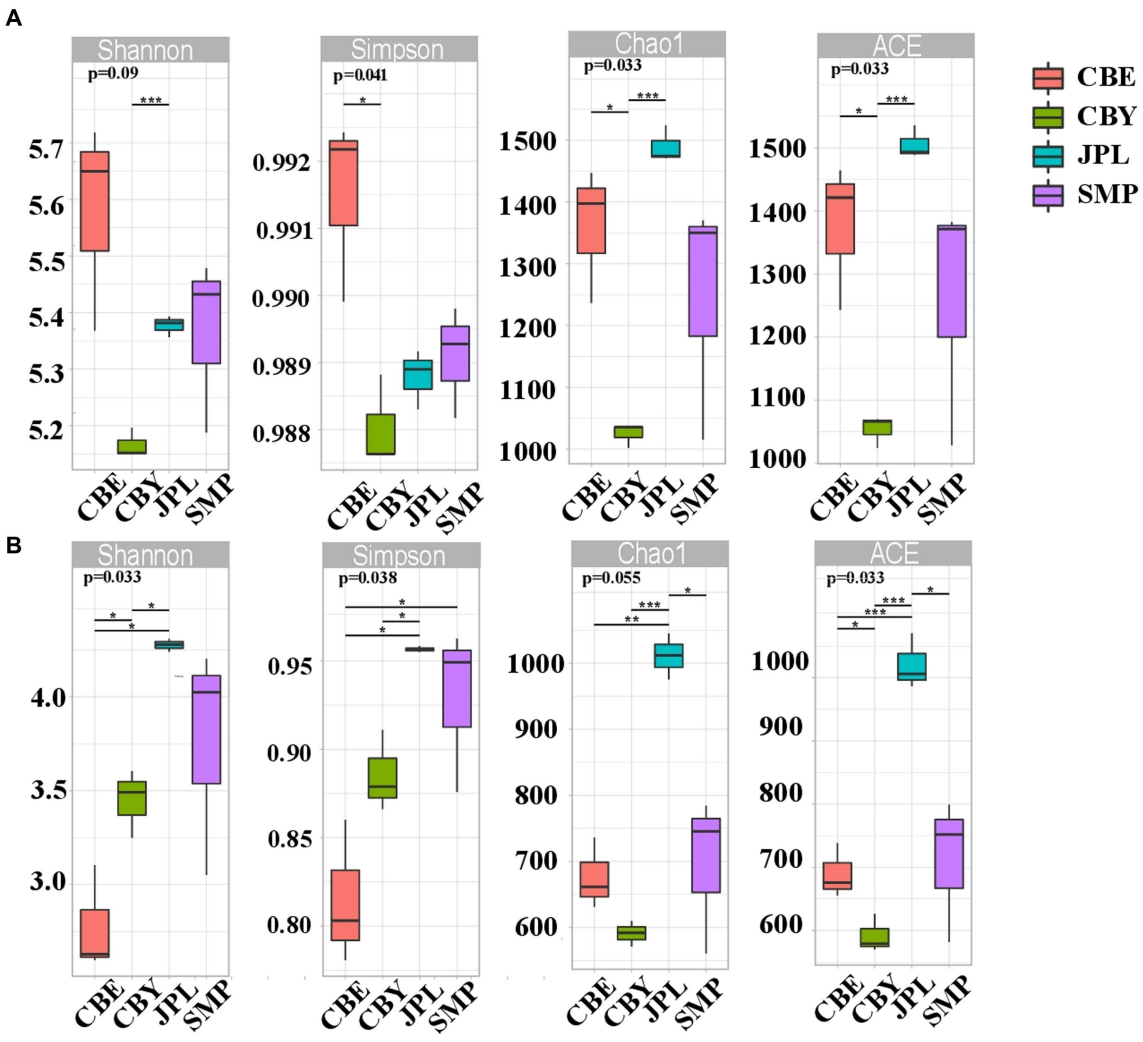
Alpha diversity of *C. argyrophylla* soil microbial communities, including community richness (observed species, Chao, ACE indexes) and diversity (Shannon, Simpson indexes). Box-and-whisker plots show the median values and their interquartile ranges. Different colors signify each sampling site. Student’s *t*-test was performed for estimators (*p < 0.05, **p < 0.01, **p < 0.001). **(A)** the Alpha diversity indices of bacterial soil microbial communities associated with *C. argyrophylla*. **(B)** the Alpha diversity indices of fungal soil microbial communities associated with *C. argyrophylla*.

The Chao1, ACE, Simpson, and Shannon alpha diversity indexes were calculated to quantify the diversity and richness of the microbial community at each site (Figure 2). The bacterial community at CBY had significantly higher Shannon indices than that at JPL (*p* = 0.09). The bacterial community at CBY had significantly higher Simpson indices than at CBE (*p* = 0.041). The bacterial community at CBE had significantly lower ACE and Chao 1 indices than those at CBE or JPL. Fungal community diversity at JPL was significantly higher than that at CBE or CBY. As expected, the bacterial and fungal communities varied at each of the different natural habitats.

**Figure 3:**
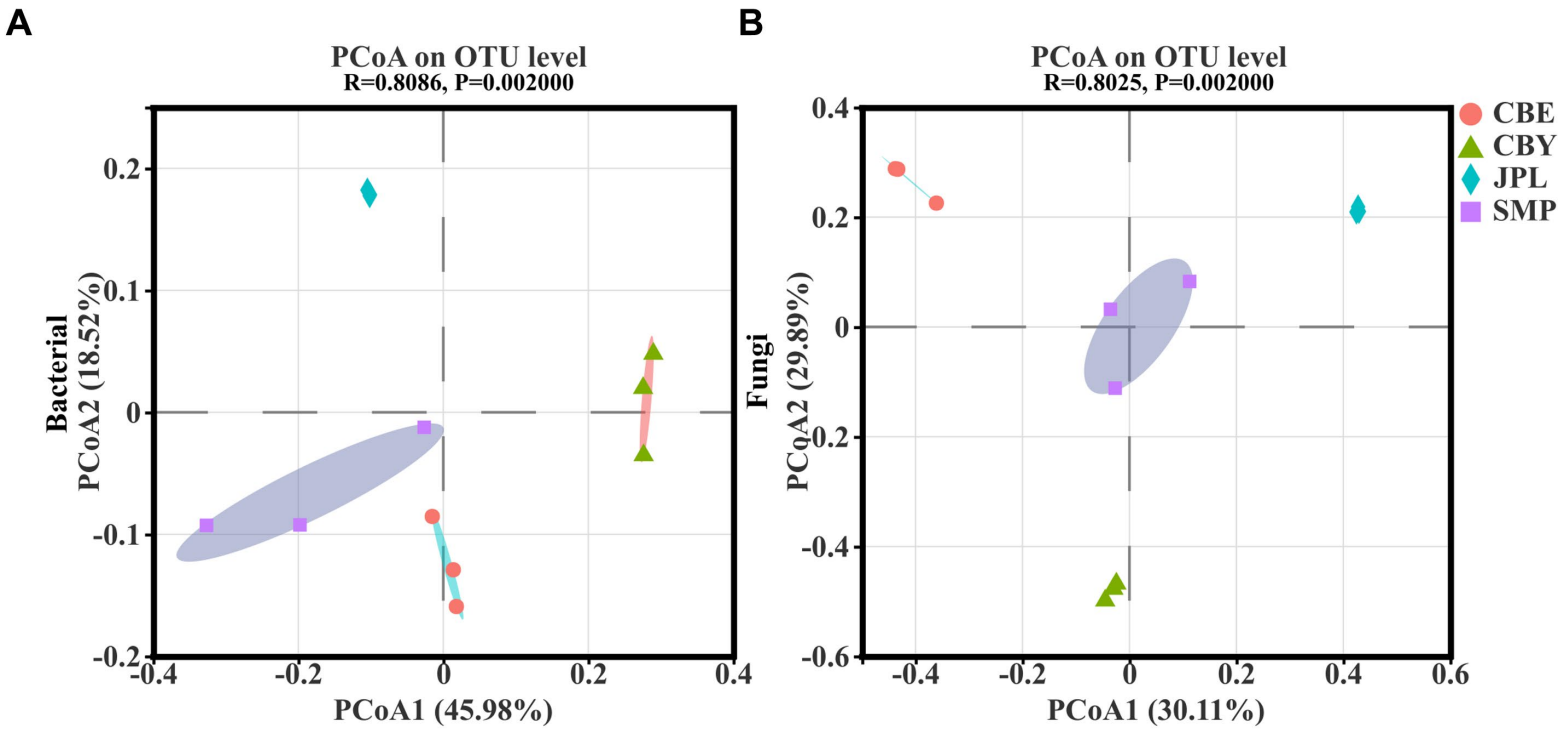
Microbial communities in the soil of *C. argyrophylla* at various locations were analyzed using principal coordinate analysis (PCoA), based on the Bray-Curtis distance. **(A)** Distribution of bacterial communities in the soil of *C. argyrophylla* at different sites. **(B)** Distribution of fungal communities in the soil of *C. argyrophylla* at different sites.

PCA of the 16S rRNA sequencing data revealed that in the bacterial community 64.5% of the variation accounted for by PCoA1 and PCoA2 (Figure 3A), while PCA of the ITS rDNA sequencing revealed that 60% of the variation accounted for by PCoA1 and PCoA2 in the fungal community composition (Figure 3B). The different replicates from each site cluster together closely. According to the 16S rRNA data, the bacterial composition was different at each location. CBY was separated from the other three groups on the *X*-axis, and JPL was separated from the other three groups on the *Y*-axis. Based on ITS gene sequencing, the fungal composition was also site-specific, with JPL clustering on the positive side of the *X*-axis and CBE clustering on the positive side of the *Y*-axis. Taken together, these findings support the hypothesis that differences in *C. argyrophylla* distribution at different natural sites may be associated with the composition of the soil microbial community.

### 3.2. Taxonomic composition of soil bacterial and fungal communities

**Figure 4:**
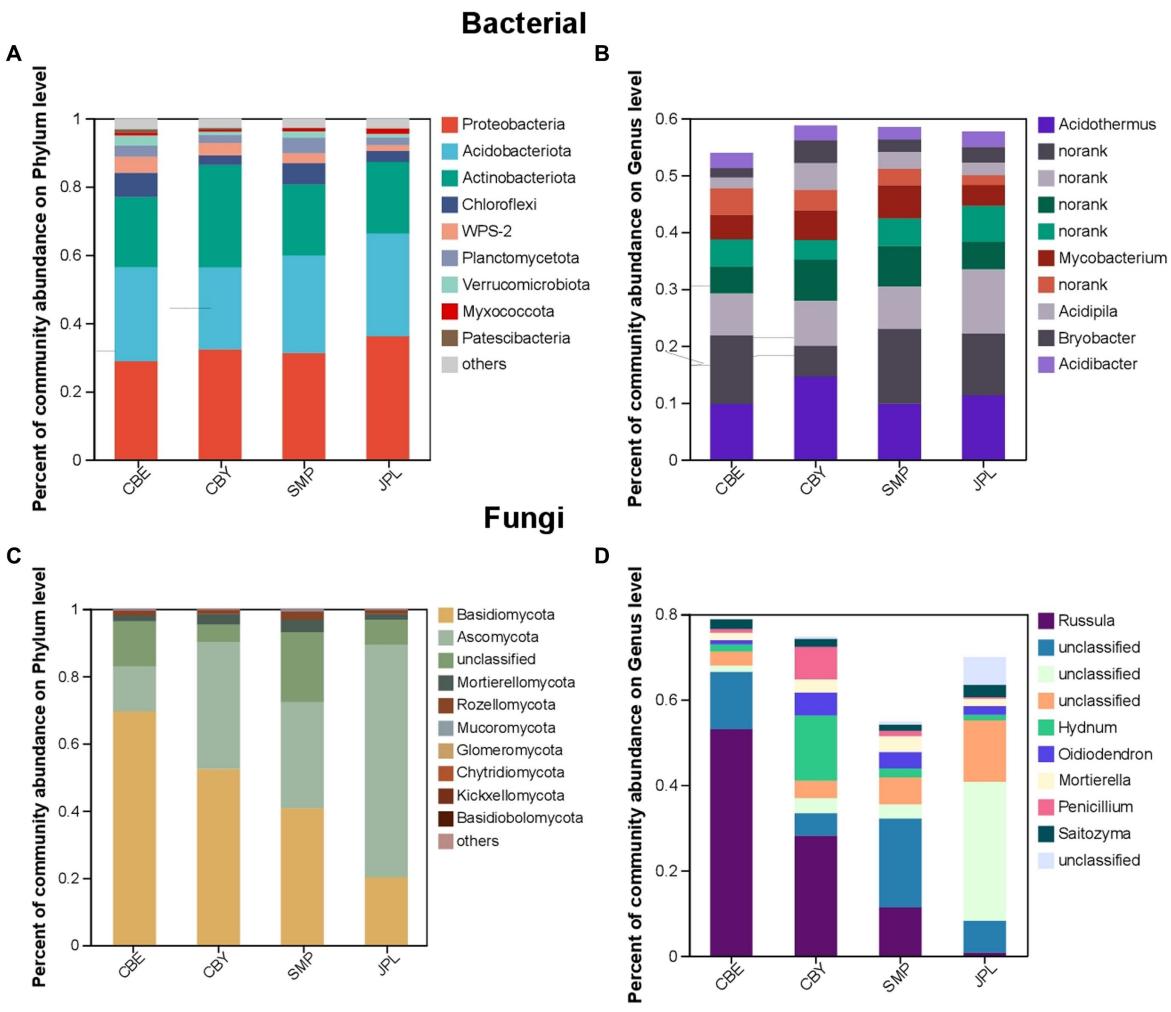
The composition of microbial communities in the soil of *C. argyrophylla* at various locations. **(A)** Phylum-level composition of the bacterial community. **(B)** Phylum-level composition of the fungal community. **(C)** Genus-level composition of the bacterial community. **(D)** Genus-level composition of the fungus community. The outermost colors represent different groups. The innermost rings represent different species. Bar width represents the relative species abundances across all samples.

The dominant major phyla in the *C. argyrophylla* rhizosphere bacterial community are *Proteobacteria, Acidobacteria*, *Actinobacteria*, and *Chloroflexi* (Figure 4A). *Acidothermus* is the most abundant bacterial genus (Figure 4B). *Basidiomycota* and *Ascomycota* were the two main fungal phyla, and were found at all sampling sites (Figure 4C). *Russula* was the dominant fungal genus (Figure 4D). These taxa were the core rhizosphere microbial taxa, and were highly abundant in the soil of *C. argyrophylla.* These core microbes may be key determinants of host health, as well as contributing to the functionality and health of the entire ecosystem.

**Figure 5:**
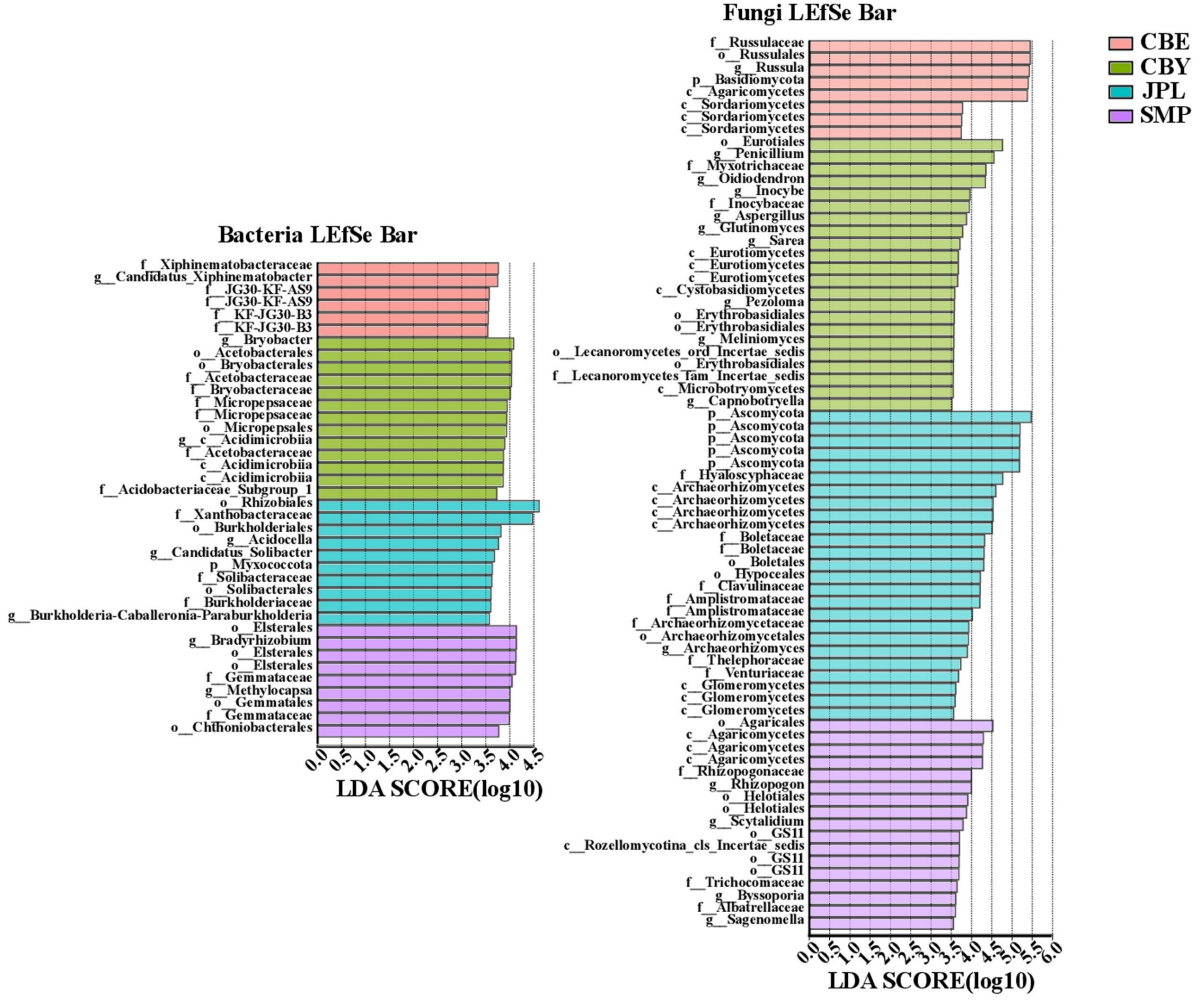
Analysis of the bacterial and fungal communities in the soil of *C. argyrophylla* at various sites using linear discriminant analysis (LDA) effect size (LEfSe) with an LDA threshold of >3.5. Groups from phylum to genus levels were determined to be significant representations of their sample group. An all-against-all comparison in multiclass analysis was performed. Each variable was ranked based on the size of its effects in each sample. When compared among samples, habitat was identified as significant (*p* < 0.05).

LEfSe was used to identify the bacterial genera with significant differences in abundance in the soil of *C. argyrophylla* at different sites (Figure 5). *Proteobacteria* was the most abundant bacterial phylum, followed by *Acidobacteria* and *Planctomycetota*. Based on these findings, the *C. argyrophylla* rhizosphere bacterial community is dominated by *Proteobacteria*, which is therefore likely to have an influence on the soil qualities”. In terms of the *C. argyrophylla* rhizosphere fungal community, the numbers of *Basidiomycota*, *Ascomycota*, *Rozellomycota*, and *Glomeromycota* differed significantly at each site. For example, *Rozellomycota* was only present at SMP, whereas *Glomeromycota* was present only in JPL, and was not significantly enriched at any of the sites. There were 22, 8, 25, and 17 enriched fungal genera in CBY, CBE, JPL, and SMP samples, respectively. *Basidiomycota* and *Ascomycota* were the two most abundant fungal phyla. In both the bacterial and fungal communities, the CBE site had significantly fewer biomarkers than the other three groups.

### 3.3. Relationship between soil microbial community structure and environmental factors

Various physicochemical characteristics of rhizospheric soil samples collected from four *C. argyrophylla* sites were analyzed (Table 2). The soil in the *C. argyrophylla* forest at each site was acidic. CBE had the highest concentrations of TN (0.7505 g/kg), TC (15.5501 g/kg), and SOM (322.6155 g/kg), while JPL had the lowest (0.4408 g/kg, 10.2801 g/kg and 123.7940 g/kg). The soil physicochemical properties at CBY and SMP were only slightly different, whereas there were larger differences in the soil properties between CBE and JPL. Specifically, JPL had much higher pH and nitrate nitrogen (NO_3_^−^-N) concentrations than the other sites, but lower nutrient concentrations.

**Table 2:** Physical and chemical characteristics (mean ± SE) of rhizosphere soil samples collected from *Cathaya argyrophylla* at different sites.

	**CBE**	**CBY**	**JPL**	**SMP**
TC (g/kg)	15.5501 ± 1.2981a	7.8576 ± 1.2099b	1.4251 ± 0.2033c	10.2801 ± 0.5471b
TN (g/kg)	0.7505 ± 0.0659a	0.3937 ± 0.0595b	0.1411 ± 0.0211c	0.4408 ± 0.0278b
SOM (g/kg)	322.6155 ± 44.2833a	191.6626 ± 24.2836b	21.2703 ± 3.1836c	123.7940 ± 8.2019b
Humus (g/kg)	1.1692 ± 0.2026a	1.2210 ± 0.08a	1.3203 ± 0.0553a	1.6890 ± 0.4371a
NH₄^+^-N (mg/kg)	69.4393 ± 4.6652a	71.0789 ± 2.5455a	15.3026 ± 2.7463c	50.7539 ± 5.9966b
NO_3_^−^-N (mg/kg)	9.8509 ± 1.0163b	2.2948 ± 0.0913b	40.6788 ± 11.7736a	1.4570 ± 0.1886b
AN (mg/kg)	184.4267 ± 6.7636a	140.3150 ± 10.9473b	40.3433 ± 6.7853d	82.9033 ± 7.5757c
AP (mg/kg)	1.1917 ± 0.0846b	1.5000 ± 0.2742b	1.1692 ± 0.0575b	2.8500 ± 0.4805a
AK (mg/kg)	94.3333 ± 10.8372a	79.000 ± 10.6927ab	47.3330 ± 21.85b	64.3333 ± 4.8074ab
pH	3.7800 ± 0.1308b	4.0933 ± 0.026ab	4.9233 ± 0.5801a	4.4233 ± 0.2718ab

Values are means ± SEs (*n* = 3). Means followed by different lowercase letters within rows are significantly (*p* < 0.05) different. TC, soil total carbon; TN, total nitrogen; SOM, soil organic matter; NH₄^+^-N; ammonium nitrogen; NO_3_^−^-N, nitrate nitrogen; AN, available nitrogen; AP, available phosphorus; AK, available potassium.

**Figure 6:**
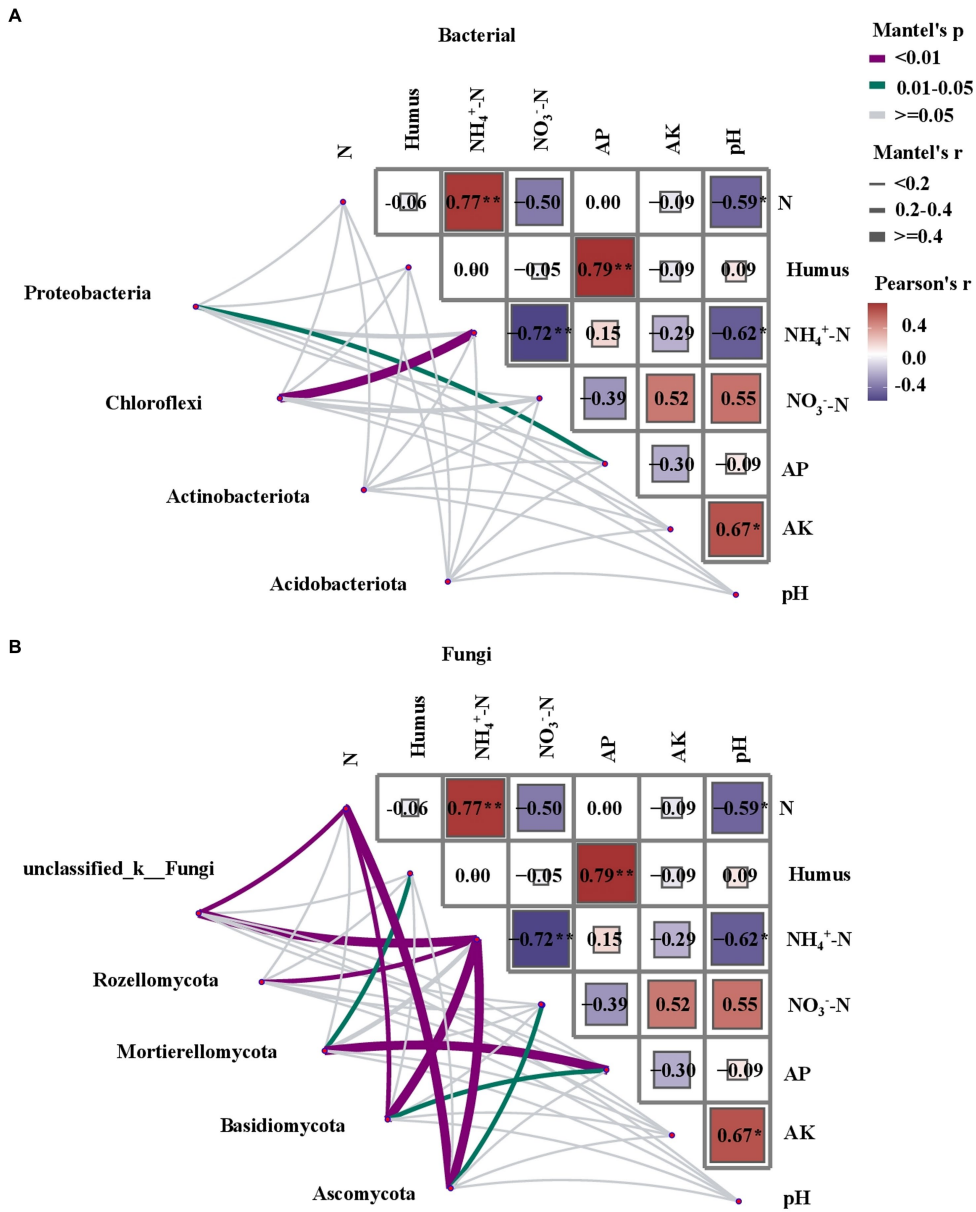
Correlation between soil characteristics and networked community structures (Bray-Curtis distance). The Mantel’s *r* value is represented by edge width, and statistical significance is indicated by edge color. The intensity of the color corresponds to the value of the Pearson’s correlation coefficient. Total nitrogen (TN), ammonium nitrogen (NH₄^+^-N), nitrate nitrogen (NO_3_^−^-N), total organic C (TOC), and pH are the soil variables. **(A)** Mantel test analysis of the relationship between bacteria and soil physicochemical properties and **(B)** Mantel test analysis of the relationship between fungi and soil physicochemical properties.

A variance inflation factor (VIF) test was performed as the first step in the empirical analysis of the soil samples, taking TN, NH₄^+^-N, NO_3_^−^-N, available P, available K, and pH into account. Mantel tests were conducted to determine the correlation between the soil microbial community structure and environmental parameters (Figure 6). There was a stronger correlation between fungal community structure and soil physicochemical properties than that of bacteria. The presence of *Chloroflexi* was strongly correlated with NH₄^+^-N (*p* < 0.05). In the fungal community, there was a significant positive correlation observed between the presence of unclassified_k_fungi, *Rozellomycota*, *Basidiomycota*, and *Ascomycota* and NH₄^+^-N (*p* < 0.01). TN was correlated with the presence of *Ascomycota*, unclassified_k_fungi, and *Basidiomycota* (*p* < 0.05). The Mantel test revealed a significant correlation between NH₄^+^-N and soil fungal and bacterial community structures, indicating that N is a major factor driving changes in soil microbial communities (*p* < 0.05).

### 3.4. Functional prediction of bacterial and fungal communities in soil

**Figure 7:**
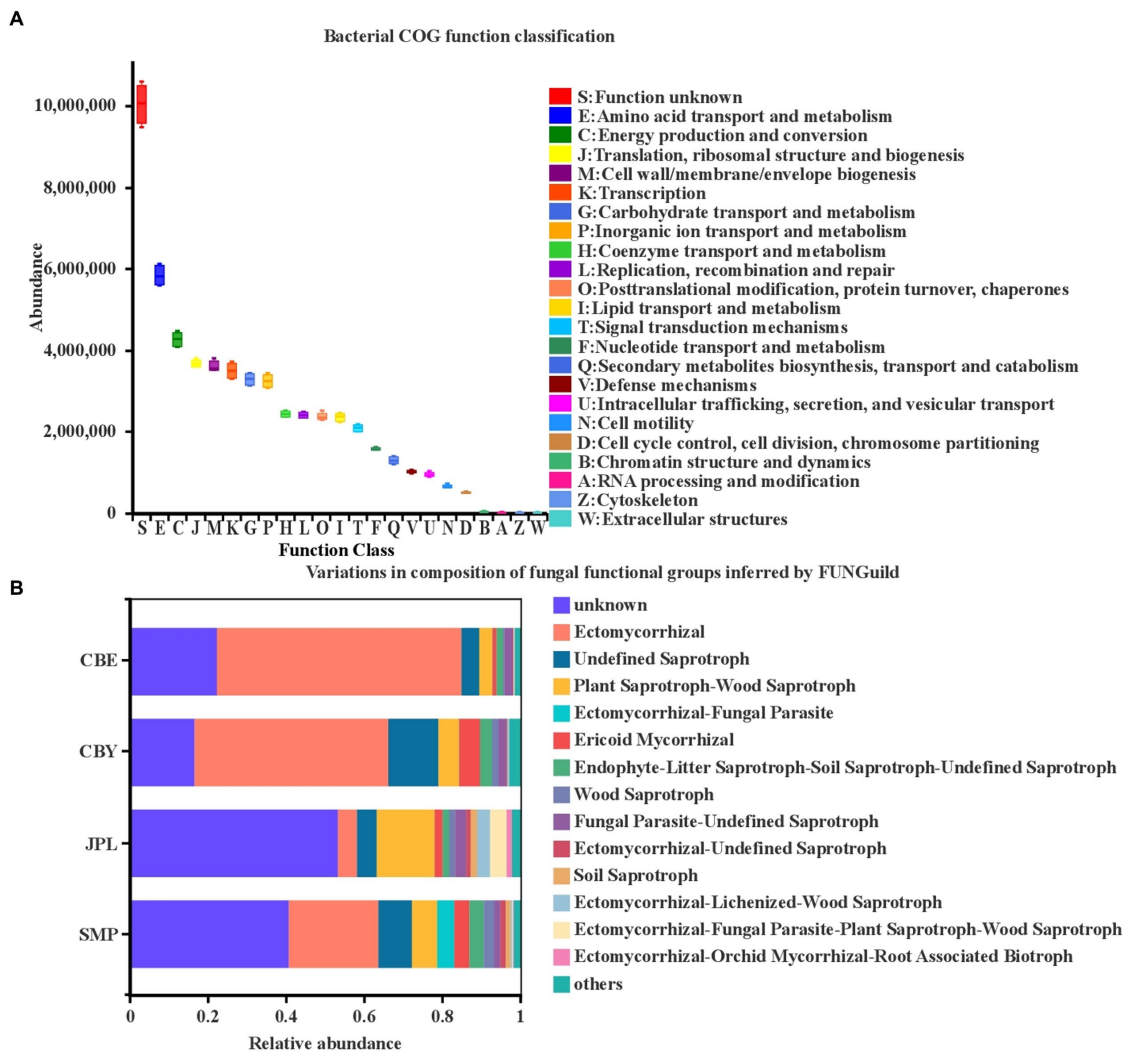
Functional characteristics of *C. argyrophylla* soil microbial communities at various locations. **(A)** Functional features of the bacterial community and **(B)** functional features of the fungal community. The *COG* classification is represented by the abscissa, and the ordinate indicates the abundance of the function.

To better understand the microecological functions of the soil rhizosphere microorganisms of *C. argyrophylla*, we used PICRUSt to assess the enriched processes associated with the *C. argyrophylla* bacterial communities (Figure 7). Among the identified taxa, functions related to bacterial metabolism were enriched, including amino acid transport and metabolism, energy production and conversion, translation, ribosomal structure and biogenesis, cell wall/membrane/envelope biogenesis, transcription, carbohydrate transport and metabolism, inorganic ion transport, and metabolism. Although the enriched functions associated with the bacterial community structure were similar at all of the sample sites, this analysis does not take the relative bacterial abundances at each site into account. The relative frequency of the major functional traits was highest for the microbial community at JPL and lowest for that at CBE.

We next separated the fungal OTUs into distinct ecological locations after assigning them to various nutritional categories. Ectomycorrhizal fungi were greatly enriched, followed by undefined saprotrophs and wood saprotrophs (Figure 7B). Ectomycorrhizal relative abundances at CBE were higher than those at the other three sites, whereas undefined saprotrophs, wood saprotrophs, and plant pathogens were the least abundant at CBE. This indicates that the broad nutritional category of the dominant fungal community in the rhizosphere of *C. argyrophylla* is different at different sites. The 2,420 fungal OTUs included 658 with a highly probable confidence ranking. These OTUs belong to eight trophic modes: *saprotroph* (42.9%), *symbiotroph* (19.3%), *saprotroph-symbiotroph* (10.3%), *pathotroph-saprotroph-symbiotroph* (10.0%), *pathotroph* (6.9%), *pathotroph-saprotroph* (6.7%), *pathotroph-symbiotroph* (3.8%), and *pathotroph-saprotroph-symbiotroph* (0.2%).

## 4. Discussion

*C. argyrophylla* is an ancient plant, and is the last remaining species in its genus. The closely-associated rhizosphere microbial community may be essential to the successful germination and growth of *C. argyrophylla* seeds. Despite this, the microbial community in the soil around the roots of *C. argyrophylla* in the wild has not been previously classified. The present study therefore aimed to use next-generation sequencing technology to survey and classify the rhizosphere of *C. argyrophylla* in order to identify soil bacterial and fungal genera associated with *C. argyrophylla* in the wild. In the present study, we explored the diversity of rhizosphere soil bacterial and fungal communities of *C. argyrophylla* across different natural distribution sites. Our results suggest that the diversity of bacterial and fungal communities may be more affected by changes in the soil environment. Previous research has demonstrated that soil nutrients are a critical factor influencing microbial communities (Bayranvand et al., 2021; Cheng et al., 2022; Xie et al., 2023), and that soil nutrients can be influenced by altitude, climate, and vegetation (Hirao et al., 2021; Zhang et al., 2022).”

In the present study, the *C. argyrophylla* rhizosphere microbial diversity may have been affected by the distinct soil physicochemical properties observed at the CBE and JPL sites, with markedly lower levels of TC, TN, SOM, NH₄^+^-N, AN, and AK detected at JPL than at CBE. We hypothesize that the differences in bacterial and fungal microbial diversity across natural distribution sites may be attributed to more intense competition for nutrients among fungal communities in areas such as CBE, where soil nutrients are more readily available, potentially resulting in an altered community structure.

Different ecological drivers can influence the composition of bacterial and fungal communities, and their responses to environmental changes are often distinct (Chen et al., 2022). Previous studies have suggested that the structure of bacterial communities is primarily determined by soil conditions, while climate is a major factor shaping fungal communities (Looby and Martin, 2020). The present findings in the natural habitat of *C. argyrophylla* support this, as we observed different bacterial and fungal communities with different soil physicochemical properties. Specifically, we found that the abundance of bacterial genera in each community was relatively stable across different sampling sites, potentially due to their greater adaptability to environmental variations compared to fungi. Conversely, the fungal community seemed to be more site-specific, suggesting that fungi are more susceptible to changes in soil composition.

At the phylum level, our analysis revealed that *Proteobacteria*, *Acidobacteria*, *Actinobacteria and Chloroflexi* are the dominant bacterial phyla at all sites. *Proteobacteria* are commonly found in nutrient-poor soils, and possess a high capacity for nutrient decomposition, allowing them to thrive in cold environments and under a broad range of growth conditions (Finn et al., 2020). *Acidobacteria* are important members of plant–soil microbiomes and agroecosystems, where they contribute to crop performance and productivity by participating in the nitrogen cycle (Kalam et al., 2020). The dominance of these bacterial phyla in the rhizosphere of *C. argyrophylla* highlights their essential roles in the decomposition of organic matter, utilization of nitrogen sources, and cycling of important nutrients such as cellulose and lignin in plant residues, ultimately promoting soil fertility. *C. argyrophylla* thrives in regions with infertile soil that is rich in *Proteobacteria*, which produce abundant nutrients. This implies that *C. argyrophylla* likely requires a substantial nutrient supply for optimal growth.

The rhizosphere fungal community composition of *C. argyrophylla* is dominated by the phyla *Basidiomycota* and *Ascomycota*, likely due to the presence of apoplastic leaf litter, which is rich in lignin and cellulose, in the growth environment of *C. argyrophylla*. These two fungal phyla play a crucial role in the breakdown of recalcitrant organic matter, particularly lignin and cellulose. Interestingly, *Basidiomycota* had the highest relative abundance in CBE, followed by CBY and SMP. The lowest abundance was found in JPL. possibly due to the more anaerobic conditions and higher lignin concentration at CBE. In contrast, the abundance of *Ascomycota* varied significantly (*p* < 0.01) across the different natural ranges of *C. argyrophylla*, with the highest abundance observed at JPL. This may be attributed to the harsh environmental conditions at JPL, where *Ascomycota*, known for their ability to adapt to environmental stress and efficiently use limited resources, play a significant role in soil organic matter decomposition.

The present study supports our hypothesis that soil properties are a critical determinant of the distribution of rhizosphere microorganisms. Previous research has demonstrated that soil physicochemical properties strongly influence soil microbial populations, and spatial variation in soil nutrients can lead to heterogeneity in fungal communities (Egidi et al., 2019; Vuong et al., 2020; Zhang et al., 2020). The combination of plant traits and soil variables explains the greatest variation in microbial communities along altitude (Ren et al., 2018; Bayranvand et al., 2021), with soil pH being particularly influential (Che et al., 2022). This is because low temperatures at high altitudes tend to reduce nutrient decomposition ability and alter plant root traits, which in turn influence the growth and diversity of rhizosphere microorganisms (Zeng et al., 2020).

Soil pH also plays a crucial role in soil bacterial and fungal communities, as minerals including K and P are more readily released and effectively used by plants in acidic environments. Additionally, soil pH can directly or indirectly influence soil properties (such as enzyme activity, C content, and nutrient effectiveness), thereby impacting the diversity and composition of soil microorganisms (Wahome et al., 2023). Plants have a preferred pH range, which is closely associated with the development of specific soil microbiota (Liu et al., 2018). In our previous study of pine forests, the sampled soils were acidic, with pH values ranging from 3.6 to 5.3 (Tian et al., 2021). The low soil pH of the growing environment of *C. argyrophylla* is likely due to the harsh conditions in which it grows, where soil sulfide complexes are oxidized or alkaline ions are leached from the soil. The soil pH at JPL was the highest among the four selected sites, and the soil bacterial community was significantly different from that of the other three sites. Hydrogen ions can affect the physiological state of plants, the structure of root secretions, and the structure of microbial communities. Soil pH is therefore an important factor to consider for the ongoing conservation of *C. argyrophylla*.

A Mantel test revealed that NH₄^+^-N and TN were the key environmental factors affecting the abundance of dominant bacterial groups in the rhizosphere microbial community of *C. argyrophylla*. Ammonium is the predominant nitrogen form in acidic or well-watered soils (Wang S. et al., 2020), and nitrifying bacteria convert ammonium nitrogen to NO_3_^−^-N, which can be readily assimilated by plants (Hachiya and Sakakibara, 2017). The distinct responses of soil bacterial and fungal communities of *C. argyrophylla* to soil physicochemical parameters may be attributed to factors such as material exchange in the soil, nutrient flow, and microbial adaptation to the environment. While soil physicochemical properties may partly account for the variation in the structural composition of regional microbial communities, preserving natural forests of *C. argyrophylla* necessitates a comprehensive and integrated technical approach. Other environmental or biological factors may also impact the community structure of *C. argyrophylla*. Future studies could explore the effects of climate, geography, soil enzyme activity, soil aggregate structure, and associated plant communities on the diversity and structure of rhizosphere microbial communities of *C. argyrophylla*. Additionally, metagenomics could be employed to identify fungi and bacteria with unknown biological functions.

In summary, the present study makes a significant contribution to our understanding of the relationship between soil properties on the *C. argyrophylla* rhizosphere, and provides valuable insights into the diversity and community structure of rhizosphere microorganisms associated with this species. Our findings underscore the critical role of soil environmental factors in shaping the microbial communities of *C. argyrophylla*, and emphasize the need for interdisciplinary approaches for the conservation and management of wild forests.

## 5. Conclusion

In the present study, we utilized high-throughput sequencing technology to investigate the composition of bacterial and fungal communities inhabiting the rhizosphere soil of *C. argyrophylla*. Our research aimed to elucidate the distribution patterns and drivers of rhizosphere soil microbial diversity in *C. argyrophylla*, thereby advancing our understanding of the rhizosphere microbes in this natural ecosystem. Our findings revealed significant differences in α- diversity and community composition of rhizosphere soil bacteria and fungi along the natural altitude gradient, with soil properties closely related to the effect of altitude on the rhizosphere soil microbial community. Specifically, we identified soil NH₄^+^-N and TN as the primary drivers of soil bacterial and fungal community changes. Importantly, we observed that the correlation between fungal communities and soil physicochemical properties was stronger than that between bacterial communities and soil physicochemical properties. The present study sheds light on the different microbial adaptation strategies of rhizosphere soil microorganisms in the natural ecosystem of *C. argyrophylla*, which are strongly influenced by soil nutrients. We also clarified the relationship between soil properties and rhizosphere soil microbial communities, thereby deepening our understanding of the rhizosphere microbial ecology of the *C. argyrophylla* ecosystem.

## Data availability statement

The datasets presented in this study can be found in online repositories. The names of the repository/repositories and accession number(s) can be found at: https://www.ncbi.nlm.nih.gov/, PRJNA902707.

## Author contributions

The main structure of the article was developed after several discussions between the co-authors. YW, KH, FX, and PX contributed to conception and design of the study. AD performed the statistical analysis. PX wrote the first draft of the manuscript. KH, AD, and FX wrote sections of the manuscript. PM, FW, and DX assisted with investigation and sampling. All authors contributed to manuscript revisions, and read and approved the submitted version.

## Funding

The study was supported by the Natural Science Foundation of Hunan Province (2023JJ30436 and 2022JJ50249), the Scientific Research Foundation of Hunan Provincial Education Department, China (20A360 and 22A0487), and the Doctoral Start-up project of Hunan University of Arts and Science (21BSQD10).

## Conflict of interest

The authors declare that the research was conducted in the absence of any commercial or financial relationships that could be construed as a potential conflict of interest.

## Publisher’s note

All claims expressed in this article are solely those of the authors and do not necessarily represent those of their affiliated organizations, or those of the publisher, the editors and the reviewers. Any product that may be evaluated in this article, or claim that may be made by its manufacturer, is not guaranteed or endorsed by the publisher.

## Footnotes

^1^ https://ccb.jhu.edu/software/FLASH/index.shtml

^2^ http://qiime.org/install/index.html

^3^ http://www.drive5.com/uparse/

^4^ https://sourceforge.net/projects/rdpclassifier/

^5^ http://www.drive5.com/usearch/

^6^ http://rdp.cme.msu.edu/

^7^ http://www.arb-silva.de

^8^ http://unite.ut.ee/index.php
